# A network that performs brute-force conversion of a temporal sequence to a spatial pattern: relevance to odor recognition

**DOI:** 10.3389/fncom.2014.00108

**Published:** 2014-09-17

**Authors:** Honi Sanders, Brian E. Kolterman, Roman Shusterman, Dmitry Rinberg, Alexei Koulakov, John Lisman

**Affiliations:** ^1^Department of Biology, Volen Center for Complex Systems, Brandeis UniversityWaltham, MA, USA; ^2^Cold Spring Harbor LaboratoryCold Spring Harbor, NY, USA; ^3^Sagol Department of Neuroscience, University of HaifaHaifa, Israel; ^4^HHMI Janelia FarmAshburn, VA, USA; ^5^Department of Neuroscience and Physiology, New York University Medical CenterNew York, NY, USA

**Keywords:** bistability, olfactory bulb, temporal sequence decoding, olfaction, receptors, N-methyl-D-aspartate

## Abstract

A classic problem in neuroscience is how temporal sequences (TSs) can be recognized. This problem is exemplified in the olfactory system, where an odor is defined by the TS of olfactory bulb (OB) output that occurs during a sniff. This sequence is discrete because the output is subdivided by gamma frequency oscillations. Here we propose a new class of “brute-force” solutions to recognition of discrete sequences. We demonstrate a network architecture in which there are a small number of modules, each of which provides a persistent snapshot of what occurs in a different gamma cycle. The collection of these snapshots forms a spatial pattern (SP) that can be recognized by standard attractor-based network mechanisms. We will discuss the implications of this strategy for recognizing odor-specific sequences generated by the OB.

## Introduction

Information that is presented sequentially is ubiquitous in the nervous system. The brain has to deal with external stimuli, such as speech, that occur over extended time periods. Furthermore, communication within the brain, such as that generated in the hippocampus (Jensen and Lisman, 1996; Lee and Wilson, 2002) and olfactory system (Shusterman et al., 2011) involves temporally extended information (see below).

Extensive theoretical work has gone into characterizing the process of recognition in attractor networks, which can classify spatial patterns (SPs) of input (Hopfield, 1982; Amit, 1993; Lundqvist et al., 2006). Moreover, there is now substantial experimental work for such networks (Rennó-Costa et al., 2014). Extended temporal sequences (TSs) could be recognized by an attractor network if they were first converted into a SP. However, there is little theoretical understanding of how a temporal-to-spatial conversion might be done. Several classes of solutions, such as the Reichardt detector (Reichardt, 1961), the tempotron (Gütig and Sompolinsky, 2006), or the time delay neural network (Waibel et al., 1989), require that the dynamics of the individual units (e.g., axonal conduction delays and membrane time constant) be on the same order as the duration of the sequence. However, these classes of solutions do not appear likely in many of these cases because the duration of sequences is >100 ms, much longer than the dynamics of single neurons, which is on the order of 10 ms.

Recent work in the olfactory system provides particularly strong evidence for the importance of TSs in sensory coding. Mitral cells of the rodent olfactory bulb (OB) generate “sharp events,” which are high-frequency bursts of action potentials (up to 200 Hz) having a duration as short as 20 ms. The sharp events evoked by a given odor in a given cell occur at a precise phase with respect to the sniff cycle (Cury and Uchida, 2010; Shusterman et al., 2011). This phase is different for different cells, tiling the several hundred milliseconds of the sniff cycle. Thus, an odor is defined by a temporally extended sequence of sharp events, similar to the odor-specific TSs demonstrated in insects (Wehr and Laurent, 1996). Importantly, the work in insects showed that the sequence is discrete because it is subdivided into packets by ongoing oscillations in the 20–30 Hz frequency range. It is likely that the temporally extended sequence observed in mammals is organized into a series of discrete packets by ongoing gamma oscillations (30–100 Hz) (Kay et al., 2009) (a result that we extend).

Here we propose a class of solutions for how the brain can recognize long sequences. We suppose that a TS produced in one area (the TS network) is transformed to a SP in a second area (the SP network). This solution relies on processes akin to working memory, a form of memory that can maintain persistent firing of a briefly presented pattern (Goldman-Rakic, 1995; Compte et al., 2000). Specifically, we propose a brute-force solution in which the SP network contains a small number of modules, each of which has persistent activity representing TS input that occurred during a specific gamma cycle. At the end of the sequence, these snapshots, collectively, would provide a SP that could be recognized by a downstream attractor network. In order to accomplish this conversion, each module in the SP network must uniquely represent the information in a specific gamma cycle within the sequence (i.e., persistent firing must not be triggered by input before or after that gamma cycle). It was therefore important to determine whether there are neurally plausible mechanisms for solving this problem.

## Results

### Discrete sequence in olfaction

**Figure 1:**
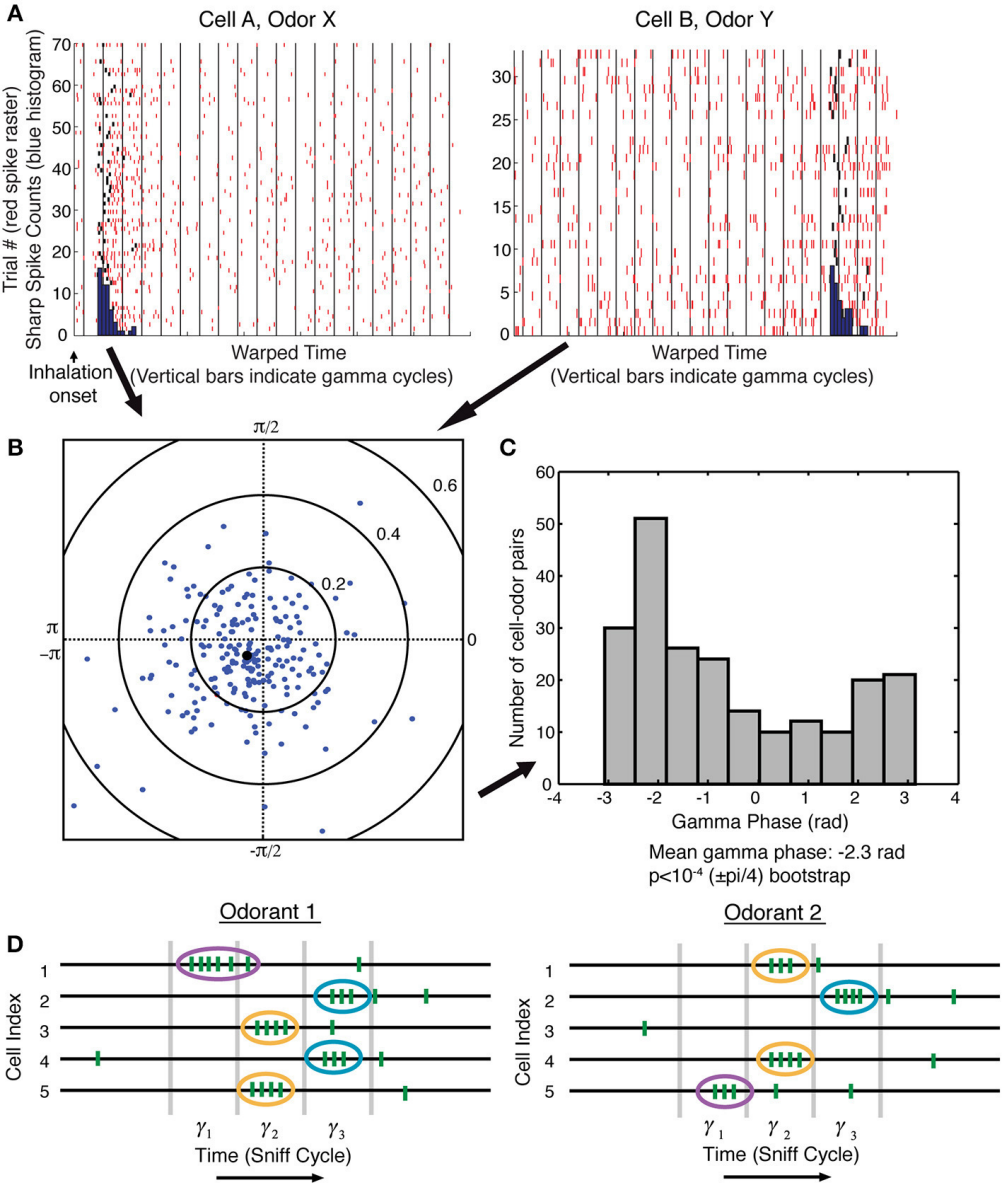
**Sharp event onsets are modulated by gamma oscillations, indicating a gamma-discretized sequence of sharp events**. **(A)** Multiple trials showing responses to an odor. Responses are shown to two cells (different odors). Red ticks are the spikes. Black ticks are spikes that were determined to be the first spike in a sharp event (see Materials and Methods). Each gamma cycle of each trial was warped so that the borders of gamma cycle are aligned with the vertical black lines. Onset histograms in warped time (blue). **(B)** The average gamma phase of sharp event onsets is plotted in polar coordinates for each of 218 cell-odor pairs. The angle of each dot represents the preferred gamma phase of sharp event onset, and the distance of the dot from the origin represents the degree of synchronization over trials. The black dot is the average over all cell-odor pairs. **(C)** Plot of phase preference for all 218 cell-odor pairs has a unimodal distribution that shows a statistically significant peaked distribution *p* < 10^−4^ (see Methods). **(D)** Schematic: sharp events (ovals) have an onset biased toward a certain phase of a gamma cycle. One can therefore think of the OB activity as a discrete sequence in which each item in the sequence is the ensemble of mitral cells that have sharp events during a given gamma cycle. Different odorants evoke different sequences of sharp events.

As a concrete example of a brain function for which this temporal-to-spatial conversion may be relevant, we turn to the mammalian olfactory system. The solution that we propose for sequence recognition assumes that the sequence is discrete. Evidence has been presented that the OB output is modulated by gamma oscillations and is therefore discrete (Kashiwadani et al., 1999). This work, however, was done before the discovery of sharp events. It was thus of interest to determine whether sharp events form a discrete sequence. We therefore analyzed sharp events identified by single-unit recordings from the OB in the awake state. The field potential was simultaneously recorded to measure gamma oscillations. Consistent with previous work (Bressler and Freeman, 1980; Kay et al., 2009), the power spectrum of the field potential showed a peak in the gamma frequency range around 60 Hz. We examined the synchronization of the onset of sharp events (time of first spike in a sharp event) with the simultaneously occurring gamma oscillations. Examining 218 cell-odor pairs (two examples shown in Figure 1A), we found that sharp event onset was modulated by gamma phase. The gamma phase of sharp event onset over the entire population was significantly biased toward a certain phase of the gamma cycle [mean gamma phase − 2.3 rad, *p* < 10^−4^ (±pi/4) bootstrap, Figure 1C]. We found no dependence of preferred gamma phase on the time of the sharp event during the sniff cycle (not shown).

This finding leads to a description of the output of the OB not as a continuous TS but, rather, as a discrete TS organized by gamma frequency network oscillations (Figure 1D). The number of such gamma cycles relevant for recognition may depend on the complexity of the recognition task, but in any case, it cannot be large, given that recognition can occur in less than a sniff cycle (Rinberg et al., 2006). For gamma cycles of about 15–20 ms, and given the fact that odor identification can often occur in less than 100–150 ms of neural processing time (Uchida and Mainen, 2003; Abraham et al., 2004), less than 10 gamma cycles define an odor sequence.

### Algorithm for sequence recognition

We now propose a brute-force mechanism for recognizing a discrete sequence organized by a series of gamma oscillations. We will formulate this mechanism in general terms, returning in the Discussion to how it might apply to odor recognition. Specifically, we propose that the SP network contains a number of discrete modules, each specialized to produce a persistent snapshot of what occurred in the TS network during a specific gamma cycle (“gamma cycle specificity”). Because these representations are persistent, a SP will evolve during the sequence as each successive gamma cycle comes to be represented by the activity in successive modules.

**Figure 2:**
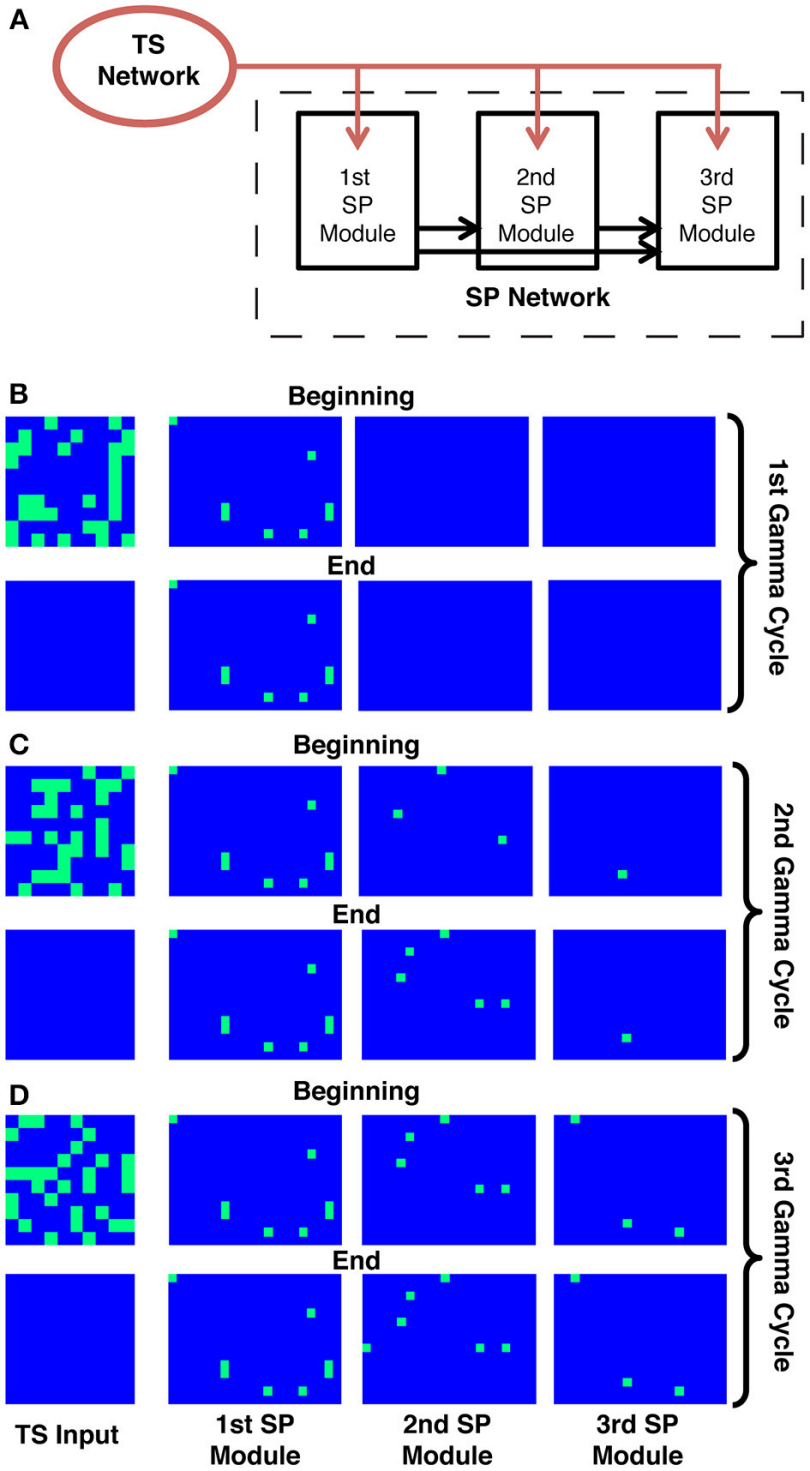
**The binary neuron model for sequence representation**. **(A)** Schematic of the model. Three distinct modules in the SP network are tuned so that they show persistent activity starting in different gamma cycles. All units receive a similar number of random connections from the TS network (red arrows). The modules have different thresholds, and the modules with lower thresholds, once activated, provide feedforward input to all modules with higher thresholds (black arrows). The first module (which has the lowest threshold) will, after activation, prime the second module, and so on. The modules also contain random recurrent connectivity among their units (not shown, see Methods). **(B–D)** Each panel shows the activity in the TS network (left) and the SP network at the beginning and the end of a given gamma cycle. Each rectangle on each panel represents the activity of a single unit, where blue represents non-firing and green represents firing. During each gamma cycle, some subset of SP cells is activated as a consequence of TS input during that cycle and fires persistently. Because of the gradient of excitability, later modules only begin to fire during later gamma cycles in the sequence.

**Figure 3:**
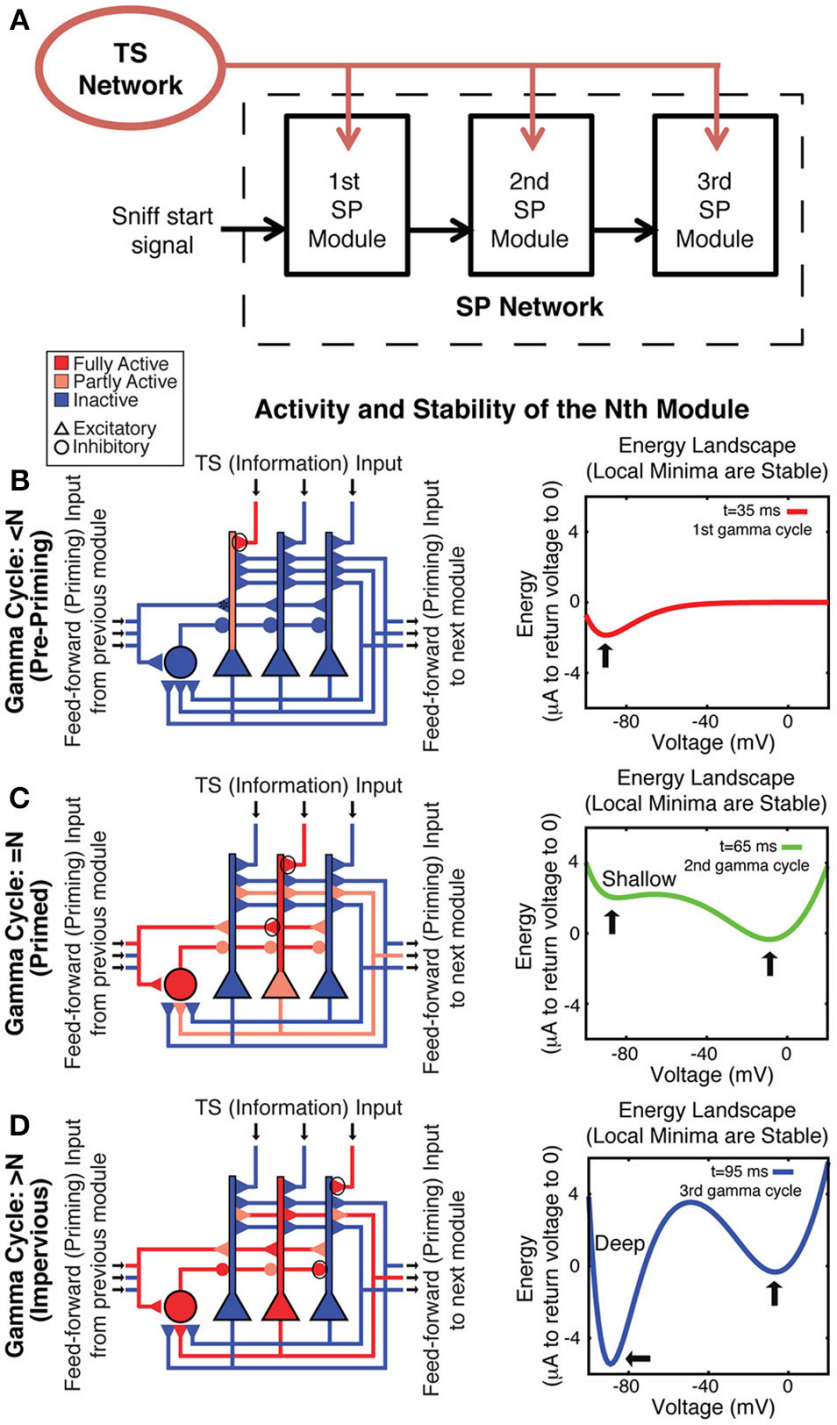
**Spiking network model architecture for sequence generation**. **(A)** TS input is provided to all SP modules. Each module receives feedforward excitatory input from the previous module onto its excitatory and its inhibitory cells, which together prime bistability in the excitatory cells. **(B–D)** On the left, a schematic of the *n*th module shows its behavior on a given gamma cycle. Three excitatory (triangle) and one inhibitory (circle) cells are shown. Blue represents hyperpolarization or lack of activity, whereas red represents depolarization or spiking. Pink represents moderate depolarization or synapses that are active but not passing much current because of their voltage dependence. On the right is an energy landscape of neuron 200 in the second module in the simulation shown in **Figure 5** to demonstrate the voltage stability of a cell before, during, and after its TS input is active during the correct gamma cycle. Local minima represent stable voltages. **(B)** Before the *n*th gamma cycle, the *n*th module has not received feedforward priming and thus is not bistable, as can be seen by the fact that there is only a single energy well in the right panel (this state is determined by leak conductance and basal KIR conductance). Any TS input during such a gamma cycle can produce firing, but this will not be maintained. **(C)** The *n*th gamma cycle is the first gamma cycle during which the *n*th module receives feedforward input and activates the NMDA conductance and slowly developing KIR (GABA-B) conductance, priming its bistability (note two stable voltages in the right panel). Because the hyperpolarized energy well is shallow, cells that receive TS input during that gamma cycle can switch to the depolarized stable state. **(D)** After the *n*th gamma cycle, slow GABA-B-activated voltage-dependent inhibition has built up enough to make the hyperpolarized state very stable (as shown by the deep energy well), preventing cells that receive subsequent TS input from switching to the depolarized stable state. Therefore, their activation is not persistent.

Two modeling approaches have been introduced for studying oscillatory networks in the brain: “generic” and “biophysical” (Skinner, 2012). We have implemented both types here. In both cases, the SP network is composed of several modules, each of which receives input from the TS network at all times (Figures 2A, 3A). Both implementations employ bistable units to produce persistent activity after appropriate activation and require “priming” by earlier modules before activating. The first model is a “generic” model network built of intrinsically bistable binary neurons, in which priming is required for the units to reach the threshold of bistability. This model, called below the binary neuron model, contains random, sparse TS-SP and SP-SP connectivity and demonstrates that TS activity of each gamma cycle becomes represented by a relatively sparse combinatorial pattern of SP neuron activation. The binary neuron model does not focus on the details of biophysical mechanism, but has the advantage of showing that the proposed sequence recognition algorithm can be implemented in a broad class of biological networks. The second model is a “biophysical” model consisting of networks of spiking neurons. In this model, neurons are not intrinsically bistable but only become bistable on receiving “priming” input from previous modules. This spiking model demonstrates the plausibility of the particular biophysical mechanism of bistability. On the other hand, the connectivity of this model is simplified by assuming one-to-one connections between the TP and SP networks such that there is no change in representation between the two networks.

### Binary neuron model

The architecture and behavior of this model are shown in Figure 2. It is assumed that the SP network is divided into a small number of ordered modules, each of which contains many neural units. During each gamma cycle, some units in the TS network are active while others are not, thus defining the input to the SP network during that gamma cycle. In addition to receiving TS input, units in each module receive excitation from active units in all previous modules (Figure 2A); they furthermore receive feedback inhibition from units in their own module (not shown). We term this the binary model because units are either active or not; for a related mechanism, see Koulakov et al. (2002). Units in each module are bistable, meaning that for some range of inputs they can be either active or inactive depending on the unit's history. Importantly, there is a threshold level of input that causes the transition from the inactive to the persistently active state. However, to reach this threshold, TS input is not sufficient in all but the first module; there must also be input from previous modules. It is assumed that bistability arises from neurally plausible mechanisms (Egorov et al., 2002; Major and Tank, 2004), but these mechanisms are not explicitly modeled. A key assumption is that, among modules, there is a gradient of the threshold required to make the transition to the active state. Part of the input needed to reach threshold can come from the active cells in earlier modules. For example, the units in the second module are initially not excited enough by TS input alone to become persistently active. However, after activation of the first module and the resulting feedforward excitation (priming), units in the second module can be persistently excited by the TS input that occurs during the second gamma cycle. The gradient of threshold enforces the requirement that TS activity before the *n*th gamma cycle not affect the *n*th module. The requirement that TS activity after the *n*th gamma cycle not affect the *n*th module is enforced by the intra-module feedback inhibition; after the *n*th gamma cycle, there is so much local inhibition that TS input is not sufficient to trigger further transitions from the down state to the up state. Cells already in the upstate can, however, continue to fire because of their bistable properties. This persistent firing thus represents a snapshot of the consequences of TS input during the *n*th gamma cycle.

Figures 2B–D shows simulations of this model. It can be seen that the input to the SP network during the first gamma cycle is retained by the first module until the end of the sequence. Similarly, what happened during the second gamma cycle is retained by the second module. This and corresponding actions in the other modules ensure that the final spatial representation depends uniquely on the TS of gamma-cycle-specific TS inputs.

### Spiking neuron model

Bistability in the above model is taken as a given without specifying a conductance mechanism. It was therefore of interest to develop a model in which bistability arises from specific conductances. To explore such models, we constructed a series of modules, each of which was composed of Hodgkin-Huxley conductance-based spiking neurons. We incorporated a previously postulated form of robust bistability arising from the interaction of NMDAR and GABA-B-activated KIR (Sanders et al., 2013). In this form of bistability, synaptic activation leads to binding of glutamate to NMDARs. However, because of the voltage dependence of NMDARs, only cells with dendrites that are already depolarized receive further depolarization, maintaining their depolarized state. On the other hand, cells with hyperpolarized dendrites receive further hyperpolarization due to the voltage dependence of the internal rectifying potassium channel (KIR) activated by the GABA-B receptor. In this model, the NMDAR conductance providing bistability is in the feedforward synapses from previous modules and in the recurrent excitatory connections within a module. The GABA-B/KIR conductance in the excitatory neurons of a given module is from that module's interneurons, which are activated by feedforward input from the previous module and excitatory units within the module. We term this “spiking neuron model.” In the binary neuron model discussed above, gamma cycle specificity is due to differing activity thresholds in the different modules. In contrast, in the spiking model, each module is biophysically identical, but sequential activation is conferred by asymmetric, feedforward activation of the NMDARs necessary for the existence of bistability.

**Figure 4:**
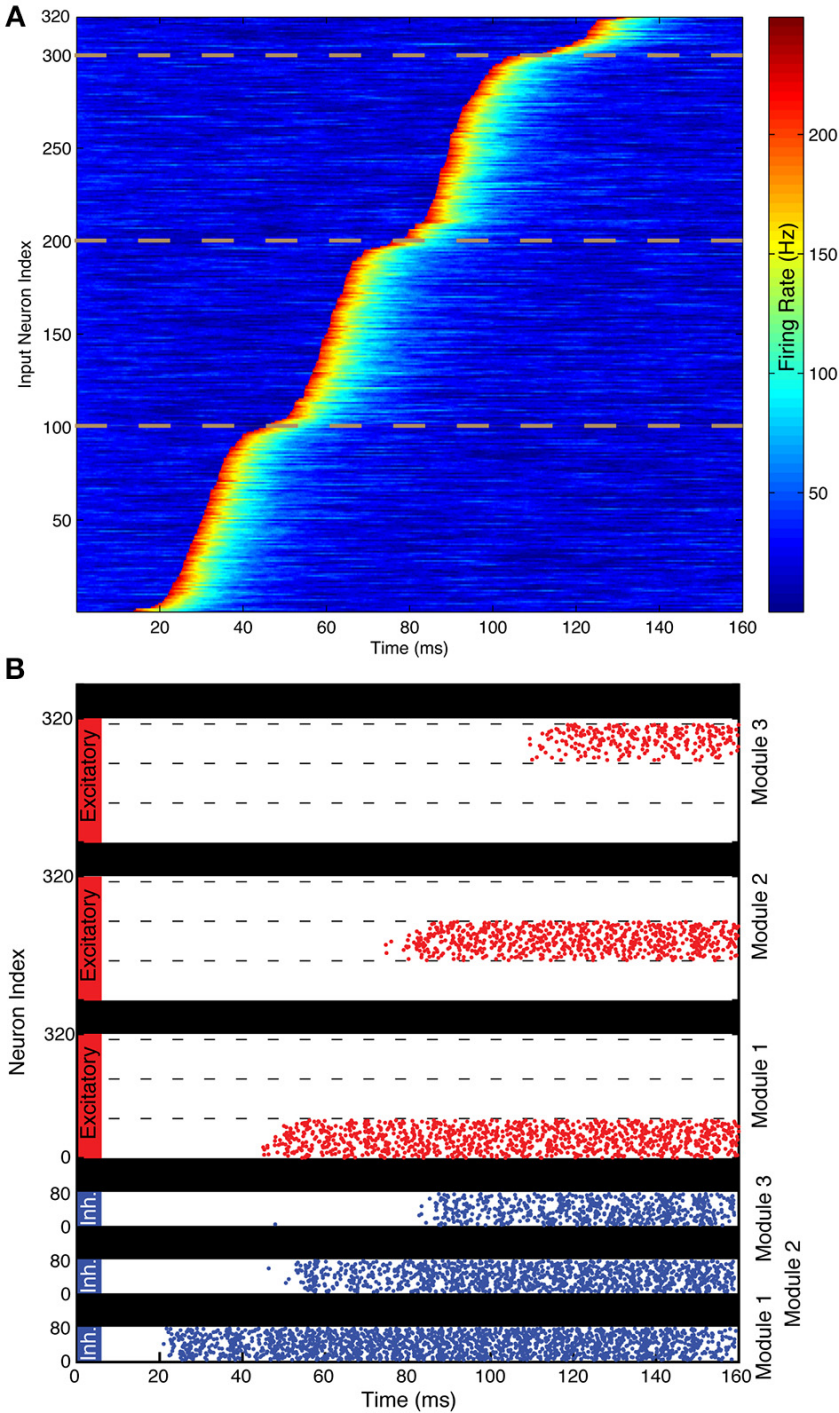
**Illustration of spiking network performance**. **(A)** Firing rate of the 320 TS cells during the simulation. Different events begin in different gamma cycles. The dotted lines demarcate the three groups of 100 cells that have events in each of the three gamma cycles shown. TS cells are ordered by event latency for ease of visual inspection. **(B)** Each excitatory cell (red) receives input from the single TS cell with the same index in this simplified simulation. Each module successfully maintains a record of the TS input that provided input during a particular gamma cycle. As shown, the 100 cells activated during each gamma cycle are sequentially numbered, but the pattern of activated cells could be arbitrary. Spikes of the 80 inhibitory interneurons in each module are shown in blue. Interneurons start spiking before excitatory neurons because of the feedforward excitation that they receive. During the last 20 ms, the SP network activity has a spatial pattern that reflects the pattern of TS cell activity during the first three gamma cycles.

Each module goes through three stages during the course of the sequence. Before the gamma cycle to which that module is dedicated (the *n*th gamma cycle for the *n*th module), there is only one stable voltage for the neurons in that module. That voltage is determined primarily by leak and constitutively active KIR. Therefore, the voltage of any neuron receiving TS input decays to rest soon after that input is removed (Figure 3B). Toward the end of the *n*-1 cycle, there will be feedforward input from the *n*-1 module that increases the AMPA and NMDA conductances on all excitatory and inhibitory cells in the *n*th module. The resulting activation of the NMDA conductance “primes” the *n*th module by creating the potential for a stable depolarized voltage in the dendrites of excitatory cells (Lisman et al., 1998). Thus, cells receiving TS input during the *n*th gamma cycle are able to switch into a stable depolarized state (Figure 3C). The feedforward projection also excites the inhibitory cells in the *n*th module, which leads to a slow increase of GABA-B-activated KIR conductance during the next 20–80 ms (Thomson and Destexhe, 1999) in the excitatory cells in the module. This eventually makes the hyperpolarized state very strongly stable (note deep energy well in Figure 3D). The strength of this stability prevents cells in the hyperpolarized state from activating due to TS input that occurs in the n+1 and later gamma cycles, locking the activity state of the module and making it impervious to further TS input (Figure 3D). Thus, the network operates in such a way that persistent firing pattern in a given module is determined by TS input only during a given gamma cycle. Figure 4 shows that a network based on these principles indeed maintains a SP that is observable at the end of the sniff and that each module represents the input that occurred during a different gamma cycle.

**Figure 5:**
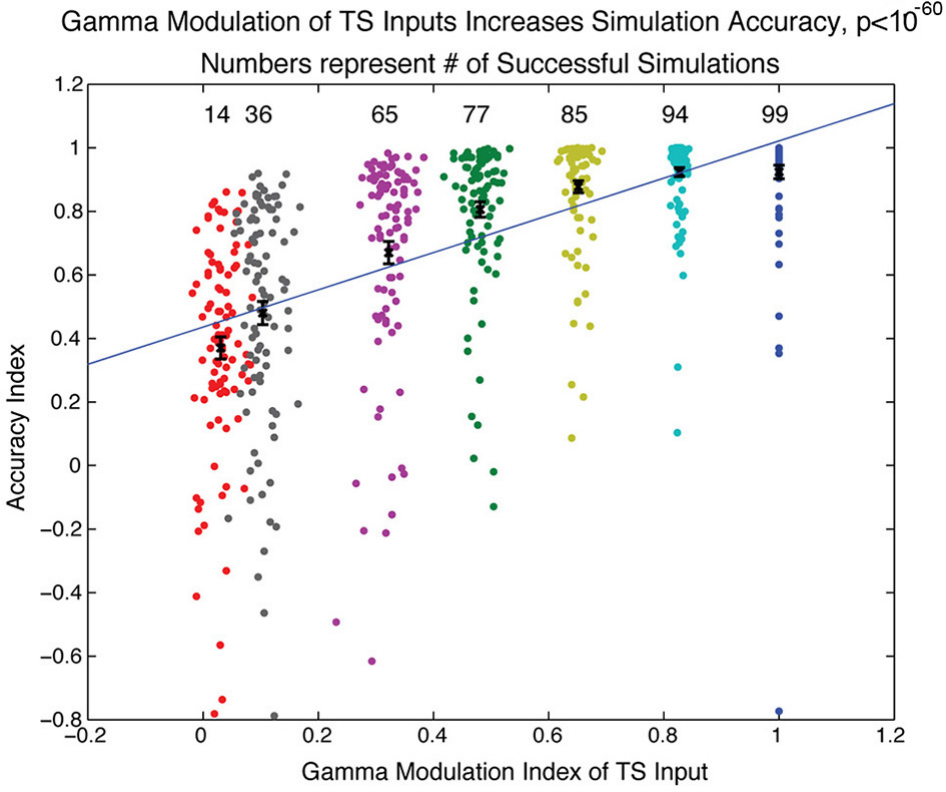
**Gamma modulation of TS input increases spiking network accuracy**. The network simulation was run over a range of parameters (see Methods) for different sharpnesses of gamma modulation of TS input to the SP network. The sharpness of gamma modulation was defined by the width of the gaussian distribution from which the timing of onset of TS cell activity was drawn for each gamma cycle. Each dot in the figure represents a simulation and is associated with the sharpness of gamma modulation (color), the measured gamma modulation index (x-axis), and the accuracy of the simulation (y-axis). The accuracy index quantified how similar the final SP network state was to the state that would be expected if each module perfectly maintained the TS input that occurred during its gamma cycle (see Methods). If a parameter set had a successful simulation (accuracy index > 0.7) for any extent of gamma modulation, the simulations with that parameter set are shown here for all sharpnesses of gamma modulation (112 total parameter sets shown). The number of successful simulations and the average accuracy index both significantly increased with increasing gamma modulation of the TS input.

Does this sequence-decoding mechanism depend crucially on the fact that TS input is discrete? To examine this question, we varied the extent of discretization of the TS and determined how this affected the accuracy of the SP produced in our SP network, quantified using an “accuracy index.” This is based on a comparison of the activity of the network in the last 50 ms of the simulation to the activity that would be expected if activity of a TS cell in a particular gamma cycle led to activity of the SP cell of the same index in the module dedicated to that gamma cycle (a positive accuracy index means that the network gets more excitatory cell activities right than it got wrong). In each gamma cycle, the variability of the TS input onset is described by the width of a normal distribution. We thus varied the extent of discretization by changing the width of the distribution of TS cell activity onsets. We ran simulations over a range of several parameters (NMDA, GABA-A, and GABA-B maximal conductance and number of TS network cells activated each gamma cycle) and plotted in Figure 5 simulations having a parameter set that was successful for at least one gamma discretization level (accuracy index > 0.7). Figure 5 shows that, as the width of the normal distribution was increased, there was reduced ability of the simulated SP network to form an accurate spatial representation of the sequence. Thus, the discretization of temporal input is important for effective decoding of sequences by a modular receiver.

## Discussion

We have presented two related models for how discrete TSs might be converted to spatial representations. In these models, there are separate SP network modules; each persistently represents information relating to TS network output during a specific gamma cycle. This gamma-cycle specificity has two requirements. One is that TS input *before* the designated cycle for that module (the *n*th cycle) does not produce persistent activity. This is solved in the binary neuron model with a gradient of threshold (for transition to the up state) in different modules requiring that all previous modules be activated before the next module can reach the activation threshold (Figure 2). In the spiking neuron model, the bistability is not intrinsic but requires feedforward “priming” of bistability by activity in the previous module before incoming TS input can be retained (Figures 3, 4). The other requirement for gamma-cycle specificity is that TS input arriving to a module *after* its designated cycle not affect activity in that module. This was achieved in both models because inhibition became strongly elevated after the *n*th cycle (Figures 2–4), preventing TS input from triggering further transitions to the up state. Our main conclusion is that there are neurally plausible ways in which network modules can produce persistent firing of the information that arrives during a specific gamma cycle. Collectively, these modules represent a collection of gamma-cycle specific snapshots of TS input, providing a complete picture of the input sequence. We term these brute-force solutions because a separate group of cells is dedicated to each gamma cycle.

An important issue is the layout of cells in such a network. At one extreme is a non-topographic model in which modules are defined completely by intra-cortical wiring among random cells. At the other extreme is topographic mapping in which all of the cells within a local region would contribute to the same module and different modules would be laid out in order. If the modules are topographically organized, this would create a cortical wave that might be detectable in the field potential (for other examples of cortical waves, see Sato et al., 2012). At this point, neither possibility can be excluded.

There have been several previous models for how a network can identify a temporally extended input sequence. One class of models depends on delays such that the beginning and end of a sequence reach a classifier at the same time (Reichardt, 1961; Tank and Hopfield, 1987; Waibel et al., 1989; Carr and Friedman, 1999; Lysetskiy et al., 2002). Our model shares the idea that the beginning of the sequence is provided to the classifier at the same time as the end of the sequence. However, in our model, the input is available to the classifier throughout the sequence so that classification can begin to occur as soon as evidence accumulates instead of having to wait until the end. Additionally, other sequence-decoding models do not employ biophysical mechanisms to generate delays of the length necessary to identify long sequences (in olfaction, these can be on the order of 100 ms). Still another class of models for sequence recognition is that of liquid/echo-state networks (Jaeger, 2001; Maass et al., 2002). These networks rely on recurrent excitation to produce a trace that lasts for longer than the input but that nevertheless decays over time. Though such a system is useful for continuous inputs, the traces produced are necessarily approximate because of decay. In contrast, the type of networks that we have analyzed can convert temporal patterns to SPs that are not subject to decay.

Our results suggest that the discretization of TSs by gamma oscillations has an important role. One function of gamma is the synchronization of inputs, which allows for linear or non-linear coincidence detection processes (MacLeod et al., 1998; Buzsáki, 2010). On the other hand, gamma discretization allows for some level of noise in the precise timing of input activity as long as spikes stay within the same gamma cycle (Shamir et al., 2009). The functions of gamma discretization have been explored in the olfactory system itself and have been shown to be of benefit in disambiguation of similar stimuli (Bazhenov et al., 2001). Our results further support this conclusion. As shown in Figure 5, increased gamma modulation of the TS input directly correlates with the accuracy of representation in the SP network. We suggest that the function of gamma demonstrated here is to take a sequence that is too long for easy continuous analysis and to divide it into discrete elements that can be easily dealt with by downstream areas.

### Application to olfaction

Extensive research has gone into the nature of temporal coding in the olfactory system (reviewed in Linster and Cleland, 2013). The rodent OB output has been shown to use a temporal code to represent odors (Shusterman et al., 2011). In fact, rats are able to perform a task that depends on the specific timing of channel-rhodopsin-induced activity relative to the sniff cycle (Smear et al., 2011), and individual neurons in piriform cortex can be sensitive to the relative timing of OB neuron activations (Haddad et al., 2013). Here we confirm that this temporal code is not continuous but is rather formed by a sequence in which sharp event onset occurs at a preferential phase of the ongoing gamma oscillation. Evidence for a similar discrete gamma code has been presented for locust olfaction (Wehr and Laurent, 1996). Thus, odor representation by a discrete TS may be a general property of olfactory recognition.

One possibility is that the conversion of the temporal code to a spatial code might occur in the first-order olfactory cortex. It is noteworthy that asymmetric, feedforward connectivity of the type assumed in our models is evident among subdivisions of olfactory cortex (Shepherd, 1998), suggestive of a functional gradient. The observed random projection from OB to PC (Stettler and Axel, 2009) is *not* inconsistent with a functional gradient because all of the modules in our model receive similar inputs However, a critical prediction of our models is that cells demonstrate odor-evoked persistent firing. Recordings from primary olfactory cortex show many cases of transient firing but few examples of persistent firing (Figure 2E of Miura et al., 2012). We emphasize that cases of transient firing do not rule out our model; this could occur when input transiently excites a module whose bistability is not yet primed by the previous module. Nevertheless, the paucity of persistent firing in piriform cortex would seem to argue that this is not the site where temporal-to-spatial conversion occurs. Another possible site for conversion of the temporal pattern to a SP is the endopiriform nucleus, which is deep to piriform cortex and is sometimes called layer 4 of piriform cortex (Shepherd, 1998). The endopiriform nucleus is a major locus of epilepsy (Demir et al., 2000), a condition that occurs when persistent firing becomes pathological. This suggests that the mechanisms of persistent firing required by our model are present in this structure. It would thus be interesting to determine whether odors can evoke persistent firing in this nucleus. If persistent firing is found, our model makes a very specific further prediction: that for a given cell, the onset of persistent firing will occur at a specific phase of the sniff cycle irrespective of the odor identity.

## Conclusion

There are many brain processes in which TSs must be processed, including hippocampal replay (Diba and Buzsáki, 2007; Jadhav et al., 2012) and language processing (Giraud and Poeppel, 2012). In psychology, the general problem of recognizing a TS and ascribing to it a single label is called chunking. Our model converts a TS to a stable SP and is thus a potential mechanism that could underlie chunking.

## Materials and methods

### Experimental procedure and ethics statement

The experimental procedure is as described in Shusterman et al. (2011).

### Sharp spiking events

Mitral cell spike trains were ordered by session and split into unit-odor pairs. The spike trains for each trial were analyzed from a four-second window centered around the inhalation onset immediately following odor presentation. The PSTH was calculated by placing the spike trains for a given unit-odor pair in 10 ms bins and averaging over trials. Unit-odor pairs were then selected for further analysis if their trial-averaged responses contained “sharp spiking events.” Sharp spiking events were defined as an increase in the PSTH with a peak at least 4.5 standard deviations (σ) above the baseline rate determined over the 2 s interval before odor presentation. The “sharp spike onset” is defined similarly to (Shusterman et al., 2011) as the time of the spike preceding the first inter-spike interval below a threshold of 1.5/maxFR (the maximum value of the PSTH) within a window around the peak of −2/maxFR to +4/maxFR. The timing of this spike was saved for each trial in the unit-odor pair's session.

### Gamma phase determination

The LFP recorded from the same electrode as the unit being analyzed was filtered in Matlab with a zero-phase, 4-pole Butterworth bandpass filter between 40 and 80 Hz to isolate the gamma oscillation. The phase of a sharp spike onset (θ) for a given trial was estimated using the LFP signal (*S*) and the timing of the first spike in the sharp event in a given trial (*t*_*sp*_) described above. The temporal length of *S* was restricted to the window around the peak of the PSTH as defined above. The phase was then calculated using θ (*t*_*sp*_) = arctan $\left(\frac{\frac{dS}{dt}{|}_{t\;=\;t_{sp}}/\sigma_{dS/dt}}{S(t_{sp})/\sigma_{S}}\right)$.

Here the time derivative of the LFP, $\frac{dS}{dt}$, was calculated using the discrete gradient function in Matlab. The mean phase angle over trials was determined using the circular mean of phases as in Fisher (1995). For each unit-odor pair, a complex number $Z=N^{-1}\Sigma_{j\;=\;1}^{N}e^{2i\theta j}$, which leads to the following results: |*Z*| is the degree of synchronization over trials with 1 being the maximum, and arg(*Z*) is the gamma phase of the sharp event onset averaged over trials. Complex variable *Z* for several cell-odor pairs is shown in Figure 1B, and the argument of *Z* is shown in Figure 1C as representing sharp event onsets.

The mean phase across unit-odor pairs can then be found by averaging the *Z*-values. To determine the *p*-value of the mean phase across unit-odor pairs, we used the random sampling with replacement method. A set of size equal to the original number of unit-odor pairs was created by randomly choosing *Z*-values of unit-odor pairs out of the original set, allowing for repeats. A new value of the mean angle was calculated from this distribution and saved. This procedure was repeated 10,000 times. The fraction of these bootstrapped mean phase angles falling outside of a window ± π /4 of the mean of the original distribution gives an estimate of the *p*-value.

### Binary neuron model (Figure 2)

To test generality of our model, we used a simple binary neuron model that included neurons with bistability in their response without specifying the origins of bistability. The model was based on random and sparse connectivity between the TS (TS, input) network and the SP (SP, modeled) network, as well as within the cortex as detailed below. Our simulation included 100 TS cells and three SP modules containing 300 neurons each. TS cells connected randomly and sparsely to cells in all of the SP modules with a 1% probability. Neurons in each SP module formed random sparse excitatory and inhibitory recurrent associative connections to other cells within their own module with 1 and 30% probabilities, respectively. SP neurons also formed random excitatory connections with the subsequent module with 1% probability. Within modules, non-zero excitatory/inhibitory connections had the following values of strengths: *W*^*ex*^_*ij*_ = 0.1 and *W*^*in*^_*ij*_ = −1.5, respectively. Non-zero projections from the TS cells were *W*_*ij*^*ex*^ = 2_, while the connections between modules were *W*^*ex*^_*ij*_ = 4.

The state of each SP neuron was defined by the input that this neuron receives *u*_*i*_ that satisfied the equation $\tau \frac{du_{i}}{dt}=-u_{i}+\Sigma_{j\;=\;1}^{N}W_{ij}^{ex}f_{j}+\Sigma_{j\;=\;1}^{N}W_{ij}^{in}f_{j}-\Delta_{i}$. Here τ = 20 is the time constant and Δ_*i*_ is the offset that determined excitability of this neuron. The offsets for the three SP modules were 0.5, 2.5, and 4.5. The activation state for each SP neuron had a hysteretic dependence on its inputs *f*_*i*_ = *F*_±_(*u*_*i*_). The activation function *F*_±_ was single valued for values of input variable *u* satisfying *u* > *u*_+_ = 1 and *u* < *u*_−_ = −10. For these values of parameters, the activation function *F*_±_ was equal to 1 and 0, respectively. Within the bistable range, i.e., for *u*_−_ ≤ *u* ≤ *u*_+_, *F*_±_ was bistable and remained constant depending on prior history. Therefore, if a neuron was activated, the activation function within the bistable range remained equal to 1, whereas for an inactivated neuron, the activation function was 0. Activation occurred when inputs exceeded *u*_+_, and inactivation happened when inputs fell below *u*_−_.

The simulation was carried out over three gamma cycles using Runge-Kutta method with time step Δ*t* = 0.2. Each gamma cycle was split into two parts lasting eight time units each. During the first part, the TS cells sent inputs to the SP network. To produce these inputs, we generated random variables *f* = 0 or 1 for each TS cell that determined its activation state. During each gamma cycle, 30% of TS cells were active. The identities of responding TS cells did not overlap between different gamma cycles. During the second part of the gamma cycle, the TS cells were silent, i.e., their activation states were zero.

### Spiking network architecture

The spiking network model is taken from Sanders et al. (2013) with minor modifications.

The networks used in this study contained three modules of *N* = 400 neurons each, of which *N*_*p*_ = 320 were excitatory and *N*_*I*_ = 80 were inhibitory. There were 320 external TS channels characterized by a firing rate, each of which synapsed onto one excitatory cell in each SP module and all inhibitory cells. Within the SP network, all excitatory neurons synapsed onto all other excitatory neurons in the same module with weight *w*_*EE*_ and onto all inhibitory neurons in the same module with weight *w*_*EI*_ as well as all excitatory neurons of the next module with weight *w*_*ffE*_ = *w*_*EE*_ and all inhibitory neurons of the next module with weight *w*_*ffI*_ = *w*_*EI*_. All inhibitory neurons synapsed onto all excitatory neurons within their module with weight *w*_*IE*_. See below for values. The first module received spiking input from 320 sources meant to represent a sequence start signal, to be described in detail later. This architecture is shown schematically in Figure 3.

### Spiking neuron model

The model neurons used in this study were Hodgkin-Huxley-type conductance-based neurons, modified from Sanders et al. (2013), who modified from Lisman et al. (1998). The excitatory neurons had two compartments: a dendrite with voltage *V*_*d*_ and a soma with voltage *V*_*s*_. Separating the spike-generating conductances from the bistable synaptic compartment allows bistability to be maintained during the large somatic voltage fluctuations associated with action potential generation.

The dynamics of the compartmental voltages and the leak, noise, AMPA, NMDA, GABA-A, and GABA-B conductances are the same as (Sanders et al., 2013) except for the following changes: (1) the maximal conductances were changed, and the ones used in this study are shown in Table 1; (2) the implementation of the external (TS) input was changed and is described below; (3) α in the equation for the NMDA dynamics was changed from 0.1 to 0.5 because NMDA conductance activation required too many spikes [α had an original value of 1 in Lisman et al., 1998 but was changed in Sanders et al., 2013 to reflect the lack of saturation of the NMDA conductance with single spikes (Popescu et al., 2004)]; (4) instead of 25% of the KIR conductance being constitutively active, 5% of the KIR conductance was constitutively active and 95% was activated by GABA-B, giving a modified equation describing the KIR current: $I_{GABA_{B}/KIR}=g_{GABA_{B}/KIR}(0.05+0.95\Sigma_{i}s_{i})\frac{V_{d}-E_{GABA_{B}/KIR}}{1+\text{exp}(0.1(V_{d}-E_{GABA_{B}/KIR}+10))}$ (see Sanders et al., 2013 for definition of all variables).

**Table 1:** **Values of maximal synaptic conductances used in simulations**.

**Synaptic conductance**	**Maximal *g* per synapse (mS/cm^2^)**		**Reversal potential (mV)**
	**Onto p-cells (excitatory cells)**	**Onto I-cells (inhibitory cells)**	
AMPA	1.125/*N*_*P*_	1.125/*N*_*P*_	0
NMDA	4.5/*N*_*P*_	0.3/*N*_*P*_	0
TS input	0.4	0.2/*N*_*P*_	0
GABA_A_	0.2/*N*_*I*_	0	−70
GABA_B_	130/*N*_*I*_	0	−90

The maximal conductance values in this table apply to the simulation shown in Figure 4. The simulations in Figure 5 all had different values of the following maximal conductances: *g*_*AMPA*_ onto p-cells and onto I-cells, *g*_*NMDA*_ onto p-cells, *g*_*GABA*_*A*__ onto p-cells, and *g*_*GABA*_*B*__ onto p-cells. The maximal conductance values used in the simulations in Figure 5 are described below under “Spiking network simulations.”

The external synaptic input to a given cell in the network *I*_*syn*,*ext*_ = *s*_*input*_*g*_*input*_(*V* − *E*_*syn*_), where *g*_*input*_ is the maximal conductance given in Table 1 and the synaptic activation *s*_*input*_ is the fraction of that conductance that is activated. *S*_*input*_ = 0.00175*r*, where the firing rate *r* of the TS input was governed by dynamics given by $\frac{\Delta r}{\Delta t}=\frac{P}{\Delta t}\delta (t-t_{sp})-\frac{r-r_{base}}{\tau_{sp}}+$ *noise*, where the peak firing rate *P* = 200 Hz, δ (*t* − *t*_*sp*_) is the Dirac delta function centered on the time of the activity peak for that TS cell, the baseline firing rate *r*_*base*_ = 20 Hz, the time constant of decay for the firing rate τ_*sp*_ = 10 ms, and *noise* is drawn independently for each time step from a normal distribution with a mean of 0 and a standard deviation of 5 Hz/Δ*t*. The factor of 0.00175 in the calculation of synaptic activation is taken from the steady-state synaptic activation: $s(r)=\frac{\tau r}{1000}\frac{\alpha \text{exp}\left(\frac{1000}{\tau r}\right)-\alpha }{\text{exp}\left(\frac{1000}{\tau r}\right)-(1-\alpha )}$, where the time constant of decay of synaptic activation τ is taken to be 2 ms and the fraction of unactivated receptor activated with a single spike α is 0.9. This function is practically linear, with a slope of ~0.00175, for firing rates <150 Hz.

For each TS cell input channel, the activity time *t*_*sp*_ = $\left\lfloor i/N_{prf}\right\rfloor \text{​}$ *t*_*gamma*_ + *noise*, where *i* is the index of the TS cell, *N*_*prf*_ is the number of cells active in each gamma cycle, ⌊⌋ is the floor function, and the length of a gamma cycle *t*_*gamma*_ = 30 ms, meaning that the first term gives the time at the middle of the gamma cycle it was supposed to be active on. *noise* is drawn from a normal distribution with mean 0 and standard deviation of 10 ms for Figure 4 and standard deviations of [0, 1.5, 3, 4.5, 6, 9, 12] in Figure 5. The TS cells were then sorted by *t*_*sp*_ and re-indexed.

As for the “sequence start signal” received by the first module, there were 320 channels, each of which projected to all excitatory and inhibitory cells in the first module with maximal synaptic conductances equal to those of the feedforward projections from module to module. Each of the 320 sources spiked at a random time taken from a uniform distribution between 15 and 30 ms after the beginning of the simulation and every 10 ms thereafter until the end of the simulation. The AMPA and NMDA synaptic activations onto all neurons in the first module from a given channel were simulated by *s*_*x*_ (*t* + Δ*t*) = *s*_*x*_(*t*)exp(−Δ*t*/τ_*x*_), and if the channel spiked during that time step, then *s*_*x*_ (*t* + Δ*t*) = *s*_*x*_ (*t*) + α_*x*_ (1 − *s*_*x*_ (*t*)), where *x* = AMPA or NMDA, τ_*AMPA*_ = 2 ms, τ_*NMDA*_ = 100 ms, α_*AMPA*_ = 0.9, and α_*NMDA*_ = 0.5.

### Spiking network simulations

For Figure 5, the simulation was run repeatedly for all combinations of the following five parameters: maximal *g*_*NMDA*,*EE*_ for excitatory-excitatory connections (both within module and feedforward from one module to the next) from the set [50, 54, 58, 62, 66, 70, 74, 78, 82, 86, 90]/(20*N*_*P*_) mS/cm^2^, maximal *g*_*GABA*_*A*__ for inhibitory-excitatory connections from the set [0.5, 1, 1.5, 2, 2.5, 3, 3.5, 4]/(10*N*_*I*_) mS/cm^2^, number of TS cell channels active on each gamma cycle *N*_*prf*_ from the set [0.10, 0.13, 0.16, 0.19, 0.22, 0.25]^*^N, maximal *g*_*GABA*_*B*__ for inhibitory-excitatory connections from the set [600, 700, 800, 900, 1000, 1100 1200, 1300]/(10*N*_*I*_) mS/cm^2^, and the standard deviation of *noise* in the calculation of *t*_*sp*_ from the set [0, 1.5, 3, 4.5, 6, 9, 12] ms. Maximal *g*_*AMPA*,*EE*_ for excitatory-excitatory connections (both within module and feedforward from one module to the next) was calculated from *g*_*NMDA*,*EE*_ by *g*_*AMPA*,*EE*_ = *g*_*NMDA*,*EE*_ /4. Maximal *g*_*AMPA*,*EI*_ for excitatory-inhibitory connections (both within module and feedforward from one module to the next) was set as *g*_*AMPA*,*EI*_ = *g*_*AMPA*,*EE*_ = *g*_*NMDA*,*EE*_ /4.

The gamma modulation index $GMI=1-\sqrt{12}\sqrt{\Sigma_{i\;=\;1}^{N}{\left(\Phi -0.5\right)}^{2}/N}$ is basically the standard deviation of TS cell activity onset phases from the cycle midpoint, normalized by $1/\sqrt{12}$ (the standard deviation from the cycle midpoint of uniformly distributed events) and subtracted from 1 in order to give an index that ranged from −1, representing all events occurring at the cycle edge, to +1, representing all events occurring at the middle of the cycle. A gamma modulation index of 0 would be arrived at with a uniform distribution of events.

The accuracy index *AI* = 1—the failure index. The failure index is calculated based on the activity of the network in the last 50 ms of the simulation. The failure index is (the number of active excitatory cells not corresponding to activity during their gamma cycle + the number of silent excitatory cells corresponding to activity during their gamma cycle) divided by the number of TS cell activities that occurred during the first three gamma cycles. An accuracy index of 1 represented that excitatory cells in the network were active if and only if their TS cell channel was active during their gamma cycle. An accuracy index > 0 meant that the network got more excitatory cell activities right than it got wrong.

Each data point in Figure 5 represents a single simulation. The simulations are colored by the standard deviation of *noise* in the calculation of *t*_*sp*_. The set of simulations that are plotted were chosen as follows. If a set of parameters [*g*_*NMDA*_, *g*_*GABA*_*A*__, *N*_*prf*_, *g*_*GABA*_*B*__] was found to give a successful simulation in any of the gamma modulation conditions (successful was defined as *AI* > 0.7), then the simulations with those parameters were plotted for all gamma modulation conditions. Of those plotted, only simulations with *AI* > 0.7 were included in the totals at the top of the graph, but all simulations plotted were included in the calculation of means, standard deviations, and regression of *AI* against *GMI*. The error bars are standard error of the mean.

Simulations were written in C++. Numerical integration was performed using Euler's method and Δ*t* = 0.025 ms. Code is available upon request.

### Conflict of interest statement

The authors declare that the research was conducted in the absence of any commercial or financial relationships that could be construed as a potential conflict of interest.
